# The Spatiotemporal Distribution and Molecular Characterization of Circulating Dengue Virus Serotypes/Genotypes in Senegal from 2019 to 2023

**DOI:** 10.3390/tropicalmed9020032

**Published:** 2024-01-27

**Authors:** Idrissa Dieng, Cheikh Talla, Mamadou Aliou Barry, Aboubacry Gaye, Diamilatou Balde, Mignane Ndiaye, Mouhamed Kane, Samba Niang Sagne, Moussa Moise Diagne, Boly Diop, Boubacar Diallo, Amadou Alpha Sall, Ousmane Faye, Abdourahmane Sow, Gamou Fall, Cheikh Loucoubar, Oumar Faye

**Affiliations:** 1 Arboviruses and Haemorrhagic Fever Viruses Unit, Virology Department, Institut Pasteur de Dakar, Dakar 220, Senegal; 2 Epidemiology, Clinical Research and Data Science Department, Institut Pasteur de Dakar, Dakar 220, Senegal; 3 Direction of Prevention, Ministry of Health, Dakar 220, Senegal; 4 Department of Public Health, Institut Pasteur de Dakar, Dakar 220, Senegal

**Keywords:** DENV, serotypes, Senegal, spatiotemporal distribution, genotypes

## Abstract

Dengue virus is becoming a major public health threat worldwide, principally in Africa. From 2016 to 2020, 23 outbreaks were reported in Africa, principally in West Africa. In Senegal, dengue outbreaks have been reported yearly since 2017. Data about the circulating serotypes and their spatial and temporal distribution were limited to outbreaks that occurred between 2017 and 2018. Herein, we describe up-to-date molecular surveillance of circulating DENV serotypes in Senegal between 2019 to 2023 and their temporal and spatial distribution around the country. For this purpose, suspected DENV-positive samples were collected and subjected to dengue detection and serotyping using RT-qPCR methods. Positive samples were used for temporal and spatial mapping. A subset of DENV+ samples were then sequenced and subjected to phylogenetic analysis. Results show a co-circulation of three DENV serotypes with an overall predominance of DENV-3. In terms of abundance, DENV-3 is followed by DENV-1, with scarce cases of DENV-2 from February 2019 to February 2022. Interestingly, data show the extinction of both serotype 1 and serotype 2 and the only circulation of DENV-3 from March 2022 to February 2023. At the genotype level, the analysis shows that sequenced strains belong to same genotype as previously described: Senegalese DENV-1 strains belong to genotype V, DENV-2 strains to the cosmopolitan genotype, and DENV-3 strains to Genotype III. Interestingly, newly obtained DENV 1–3 sequences clustered in different clades within genotypes. This co-circulation of strains belonging to different clades could have an effect on virus epidemiology and transmission dynamics. Overall, our results highlight DENV serotype replacement by DENV-3, accompanied by a wider geographic distribution, in Senegal. These results highlight the importance of virus genomic surveillance and call for further viral fitness studies using both in vitro and in vivo models, as well as in-depth phylogeographic studies to uncover the virus dispersal patterns across the country.

## 1. Introduction

Dengue fever (DF) is recognized as the most widespread arboviral disease globally [1]. Its transmission occurs both in urban and sylvatic cycles [2]; the main transmission cycle (urban) takes place when infected mosquito vectors from the *Aedes* genus bite humans [3]. Many factors, including climate change, increasing travel and trade, and urbanization [3] contribute to the disease spread worldwide. According to World Health Organization estimates, approximately 3.6 billion people worldwide are at risk of dengue infections [4], and reported cases range from 50 to 100 million annually [1]. Tragically, the disease results in an estimated 10,000 deaths each year [2]. Infection with dengue virus (DENV), can manifest in various clinical forms, ranging from a self-limited disease to the life-threatening severe dengue [5].

In Africa, the virus was thought to be rare; however, detection from returning travelers to other countries [6,7] and recently reported outbreaks highlighted the virus’s circulation in the continent [8,9]. Due to the lack of sufficient diagnostic tools and effective surveillance, the true burden of DENV infection is likely to be underestimated [7,10].

In Senegal, the first dengue case was reported in 1970, and the spread of the disease was mainly dominated by the occurrence of the sylvatic cycle up to the 2000s [11]. The first urban dengue epidemic took place in 2009 and was caused by DENV-3. This was followed by yearly outbreaks affecting different regions of the country [12,13,14] and linked to different serotypes [12,15,16]. Despite the recurrent reports, few studies have assessed the virus diversity in Senegal [15].

Antigenically, DENVs are categorized into four distinct serotypes, namely, DENV-1, DENV-2, DENV-3, and DENV-4, all inducing only limited cross-protection immunity [17]. Serotypes share 65–70% of amino acid sequence conservation [18]. Furthermore, each serotype is subsequently subdivided into various genotypes, which follow a marked geographic distribution [17]. Within each serotype, genotypes are defined by group of viruses sharing less than 6% nucleotide divergence and up to 9% for DENV-2 [18]. Antigenic differences among serotypes and genotypes play a crucial role in dengue epidemiology and pose challenges for vaccine development and disease control strategies [18,19].

Therefore, it is essential to engage in monitoring strains that are prevalent in specific regions. This surveillance will inform the choice of suitable prophylactic and preventive actions [9]. Additionally, diverse DENV serotypes or genotypes have been observed to elicit distinct immune responses. This variance influences their capacity to infect particular target cells and can cause more severe manifestations of dengue virus disease or differentially infect mosquitoes [20,21,22].

Although reports of dengue circulation in Africa exist, there are only a few studies that have examined the genetic makeup of the prevalent strains, focusing on both the serotype and/or genotype [23,24,25].

Dengue strains in Africa remain poorly characterized, with African sequences representing <1% of global sequence data [26]. Existing sequence data are mainly obtained during outbreak periods (e.g., in Burkina Faso or Senegal) or from returning travelers [7]. In contrast, many studies in Asia [27,28,29,30] and the Americas [31] were focused on the genetic diversity of circulating DENV strains.

Due to the unprecedented and growing numbers of confirmed DENV cases in Senegal [15] and the co-circulation of different viral serotypes [29], continuous monitoring of circulating virus variants (serotypes/genotypes) and genomic surveillance of viral strains appear to be pivotal to anticipate worsening situations. Herein, to obtain insights into the circulating DENV serotypes/genotypes and understand their spatial and temporal distribution across the Senegal, we combined epidemiology, RT-qPCR, and genome sequencing to uncover the viral genetic diversity from January 2019 to February 2023.

## 2. Materials and Methods

### 2.1. The Febrile Illnesses Surveillance System

The increasing threat of emerging pathogens of public health importance requires a reliable surveillance system to control their spread. In Senegal, the Institut Pasteur de Dakar (IPD) partnered with the Senegalese Ministry of Health and the WHO country office to implement a nationwide Syndromic Sentinel Surveillance System called the 4S network in 2011 [32]. Initially, the surveillance was limited to virologic surveillance of Influenza-like Illness (ILI). In 2015, it was expanded to include a wider range of pathogens that are associated with public health priority syndromes such as malaria, dengue-like syndromes, and diarrheal syndromes. This syndromic approach enables the early detection of unexpected and/or unusual occurrences of specific symptoms to monitor the evolution of the diseases under surveillance, investigate outbreaks, and implement appropriate response actions. The network has up to 20 sentinel sites distributed across the 14 administrative regions of Senegal, selected based on the WHO-recommended attributes [33]. The suspected DENV samples tested during this study were collected throughout the 4S network system.

### 2.2. Sample Shipping to WHO Collaborating Center for Arboviruses and Hemorrhagic Fevers at Institut Pasteur de Dakar

On a weekly basis, suspected blood samples collected from sentinel sites were transiently stored at +4 °C while awaiting shipping with clinical and demographic forms at the virology department at the Institut Pasteur de Dakar. At IPD, samples were subjected to molecular screening for the detection of 7 medically important arboviruses including Dengue, Zika, Yellow Fever, Chikungunya, Rift valley fever, West Nile, and Crimean Congo Hemorrhagic fever viruses.

### 2.3. RNA Extraction

Blood samples from suspected dengue cases were subjected to centrifugation at 2000 rpm for 5 min to obtain sera, which were harvested and aliquoted on 2 mL cryotubes for immediate use and further biobanking. RNA extraction was performed using 140 µL of sera using Qiagen viral RNA mini kit according to the manufactures’ recommendation. RNA was eluted to a final volume of 60 µL and conserved to −80 until further use.

### 2.4. RT-qPCR DENV Detection

The presence of DENV RNA from extracted RNA was assessed using RT-qPCR with sets of primers targeting 3′-UTR region of all dengue serotypes [34]. Reactions were performed as follows: 50 °C—10 mn, 40 cycles of 95 °C—1 mn; 95 °C—15 s and 95 °C—30 s. All samples with a Ct value below the fixed cut-off value of 32 were considered DENV+.

### 2.5. DENV Serotyping

Serotypes of DENV+ samples were assessed by RT-qPCR according to a protocol previously described by Dieng and colleagues [35]. Briefly, the CDC dengue typing kit [36] was used according to the manufactures recommendations. The system allows for the simultaneous detection of DENV serotypes from 5 µL of input RNA. Each of DENV serotypes can be read in different dye channels.

### 2.6. cDNA Synthesis and Amplicons Generation

To maximize yield and genome coverage, a subset of DENV+ RNA samples with Ct values < 30, maximizing spatial provenance and year of collection, was chosen for sequencing. For selected samples, cDNA synthesis was carried out using the Luna Script RT SuperMix (5X) from New England Biolab, Ipswich, MA, USA. In brief, 8 µL of RNA was mixed with 2 µL of master mix, pipetted up and down up to ten times, and briefly centrifuged. The mixture was then incubated at 25 °C for 2 min, 55 °C for 20 min, and 95 °C for 2 min, and finally placed directly on ice until further use. Then, according to the serotype, a specific whole-genome multiplex PCR was conducted in order to amplify the entire coding region of DENV using two primer pools (1 and 2) in separated tubes. Reaction conditions were previously described by Dieng and colleagues [37]. Amplification success was checked at the end of the reaction by agarose gel electrophoresis.

### 2.7. Library Preparation and Sequencing

Amplicons were purified using 1× Ampure XP Beads (Beckman Coulter Inc., Brea, CA, USA) and cleaned-up concentrations of each PCR product were measured using a Qubit dsDNA HS Assay kit (Thermo Fisher Scientific, Waltham, MA, USA) on a Qubit fluorometer (Thermo Fisher Scientific). Targeted whole-genome sequencing of DENV 1–3 was undertaken for each sample; equal concentrations of pool 1 and pool 2 amplicons were pooled per sample before library preparation using Illumina DNA preparation kit Nextera DNA flex (Illumina Inc., San Diego, CA, USA) according to manufacturer’s recommendations. Whole-genome sequencing was performed with paired-end reads using Illumina MiSeq reagent kit V3 (300 cycles) on an Illumina MiSeq instrument. Consensus sequences of around 10 Kb (corresponding to the full CDS) were generated by de novo assembling using Genome Detective (https://www.genomedetective.com/app/accessed on 15 February 2023).

### 2.8. Dataset Construction and Phylogenetic Analysis

Identification of DENV serotypes/genotypes/lineages was performed using dual procedures:

(i) By using the Genome Detective dengue typing tool;
(ii) By using a maximum likelihood (ML) phylogenetic analysis to put newly sequenced DENV strains in a global context and explore the relationship with other available global sequences.

For this purpose, we retrieved dengue virus genome sequences from US National Institutes of Health National Institute of Allergy and Infectious Diseases Virus Pathogen Database and Analysis Resource (http://www.viprbrc.org accessed on 15 February 2023) representative sequences of described dengue genotypes for each serotype. Downloaded datasets for each serotype (DENV-1, n = 202; DENV-2, n = 257, DENV-3, n = 133) contained all genomes from Africa and ≈10% of the remaining genomes. Full details of used sequences can be found in Table S1. Multiple sequence alignment was performed using MAFFT version 7.455 [38] and then manually curated to remove artefacts with AliView version 1.26 [39]. Maximum likelihood (ML) trees were generated using IQ-TREE software version 1.5.5 [40] under appropriate models, which were inferred as best fit models for DENV 1–3 by ModelFinder application implemented in IQ-TREE software version 1.5.5 [41]. Robustness of tree topologies was determined using 1000 replicates. Tree visualization was performed using Figtree (http://tree.bio.ed.ac.uk accessed on 26 February 2023) and ggtree package implemented in R software version 4.3.0 [42].

### 2.9. Temporal Trend and Spatial Mapping of Detected Serotypes

Temporal and spatial mapping of detected DENV serotypes was performed using the epidemiological week of sampling and information related to the latitude and longitude of sentinel sites from which samples were collected. Temporal trends were represented using a barplot, and spatial distribution was represented by a map made using maplots package within R; a pie chart representing the proportion of each detected serotypes at a given region was generated.

## 3. Results

**Figure 1:**
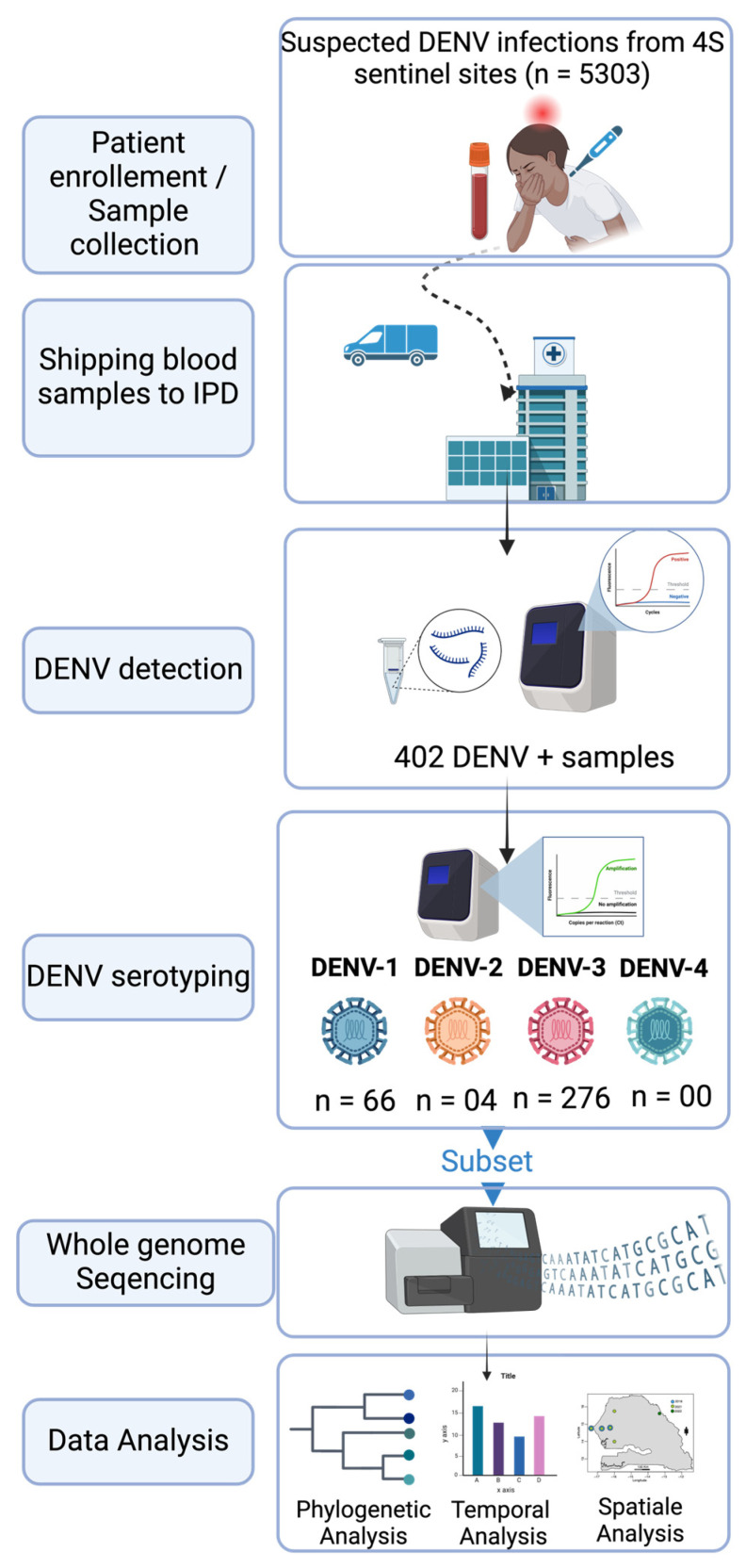
Used workflow during this study.

From January 2019 to February 2023, 5303 suspected arboviral cases were collected from 4S sentinel sites and shipped to WHOCC for arboviruses and hemorrhagic fever virus molecular testing for the detection of DENV RNA. A total of 402 samples were panDENV-positive according to RT-qPCR (Figure 1).

**Figure 2:**
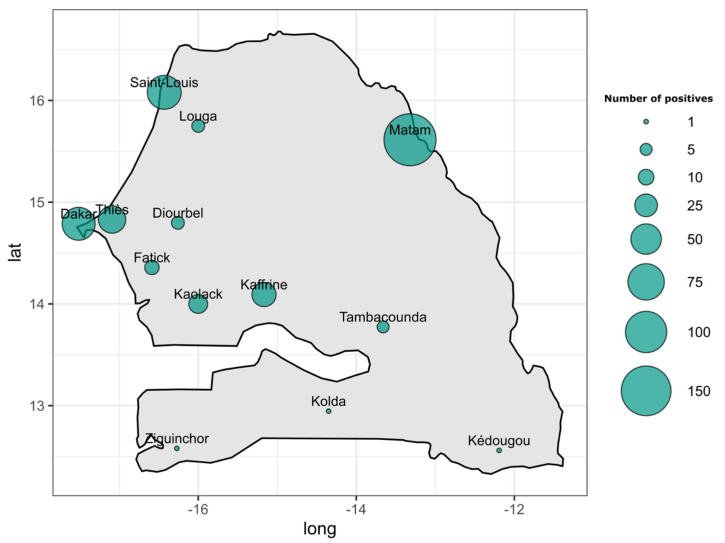
Map showing the spatial repartition of DENV RT−qPCR-positive samples collected between January 2019 and February 2023. The size of the dot is proportional to the number of recorded dengue-positive cases in each administrative region of Senegal.

According to the region of provenance, RT-qPCR DENV+ samples were collected from twelve out of fourteen administrative regions of Senegal. In term of occurrence, the highest numbers of confirmed DENV+ cases were recorded in Matam (n = 166), Saint louis (n = 64), Dakar (n = 60), Thies (n = 39), Kaffrine (n = 29), and Kaolack (n = 16). Remaining regions including Diourbel, Fatick, Kedougou, Kolda, Louga, Tambacounda, and Ziguinchor all recorded less than ten confirmed DENV+ cases (Figure 2 and Table S1).

Briefly, the highest number of confirmed dengue cases was obtained in 2022 (n = 216). The lowest number of suspected (n = 109) and confirmed cases (n = 15) were obtained during the year 2023.

Serotyping results by RT-qPCR of 346 out of 402 DENV+ samples showed the circulation of three DENV serotypes.

**Figure 3:**
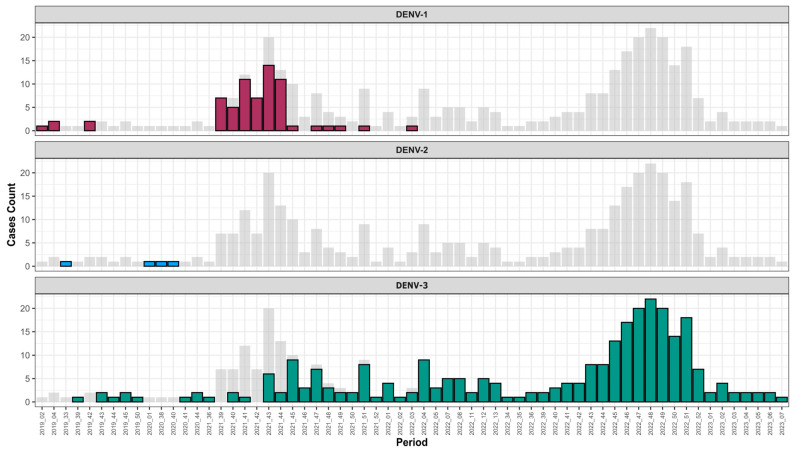
Pattern of DENV serotypes circulation in Senegal through the 4S network from 2019 to early 2023; for 2023, only surveillance data between January and February were included. Numbers of positive are represented on a weekly basis; serotypes are colored as follows: DENV-1 in red, DENV-2 in blue, and DENV-3 in green. Grey background represents the total of serotyped samples per week.

In terms of occurrence, DENV-3 was the most prevalent serotype (n = 276), followed by DENV-1 (n = 66); the less prevalent serotype among screened DENV+ samples was DENV-2 (n = 4) (Table S2). Interestingly, temporal distribution of circulating serotype during the study period showed that between February 2019 and March 2022, the three serotypes that co-circulated after March 2022 DENV 1–2 were no longer detected, and only DENV-3 circulated until February 2023 (Figure 3 and Tables S3 and S4).

**Figure 4:**
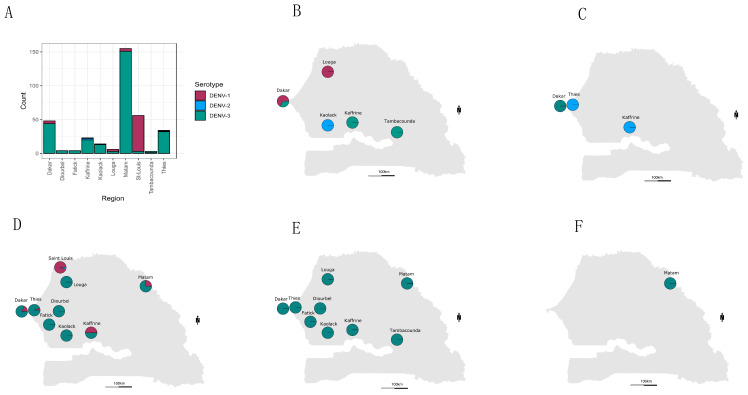
(**A**) Barplot showing the number of detected serotypes per region. (**B**–**F**) Maps showing the spatial repartition of detected dengue serotypes from 2019 to early 2023 (up to week 07 of year 2023). Pie charts for each sampling region display the proportion of serotyped samples by RT-qPCR. DENV-1, DENV-2, and DENV3 are colored, respectively, in red, blue, and green. The size of the circle is not proportional to the number of cases. A summary of serotyped sample numbers and results for each monitoring region can be found in Table S1.

The most widely distributed serotype was DENV-3, which was found in ten out of ten regions where serotyped samples were retrieved. The highest numbers of DENV+ cases associated to this serotype were found in Matam (n = 151), Dakar (n = 44), and Thies (n = 32). DENV-1 was detected in four regions including Saint-Louis (n = 53), Dakar (n = 4, Matam (n = 4), Louga (n = 3), Kaffrine (n = 1), and Thies (n = 1). Finally, DENV-2 was the least detected serotype, with the detection of only 4 cases in Kaffrine (n = 2), Kaolack (n = 1), and Thies (n = 1) (Figure 4 and Table S2).

The DENV typing tool results (Table 1) classified DENV-1 sequences into genotype V, DENV-2 into the cosmopolitan genotype, and finally, DENV-3 as genotype III.

**Figure 5:**
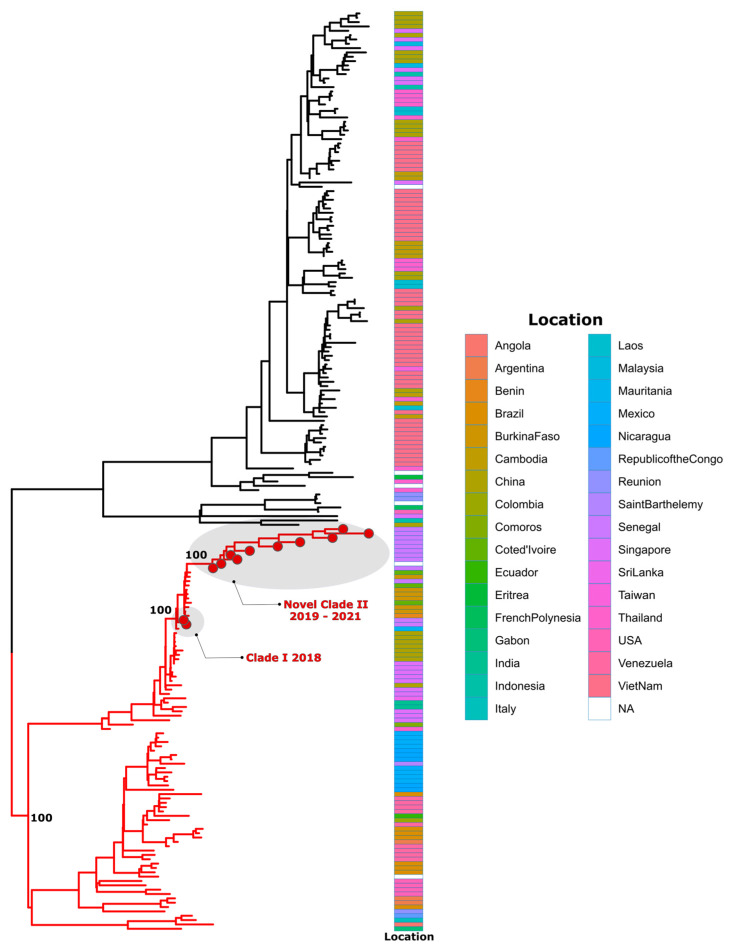
Maximum likelihood (ML) tree of DENV–1 in Senegal from January 2019 to February 2023. Branches of sequences belonging to genotype V are colored in red. The analysis was based on 11 nearly complete genomes of DENV–1, generated during this study, in addition to n = 202 available sequences that were retrieved from VIPR database. The tree is midpoint-rooted. Study sequences harbor points on the sequence tip. Heatmap represents the location of collection of used DENV-1 sequences during phylogenetic analysis.

**Figure 6:**
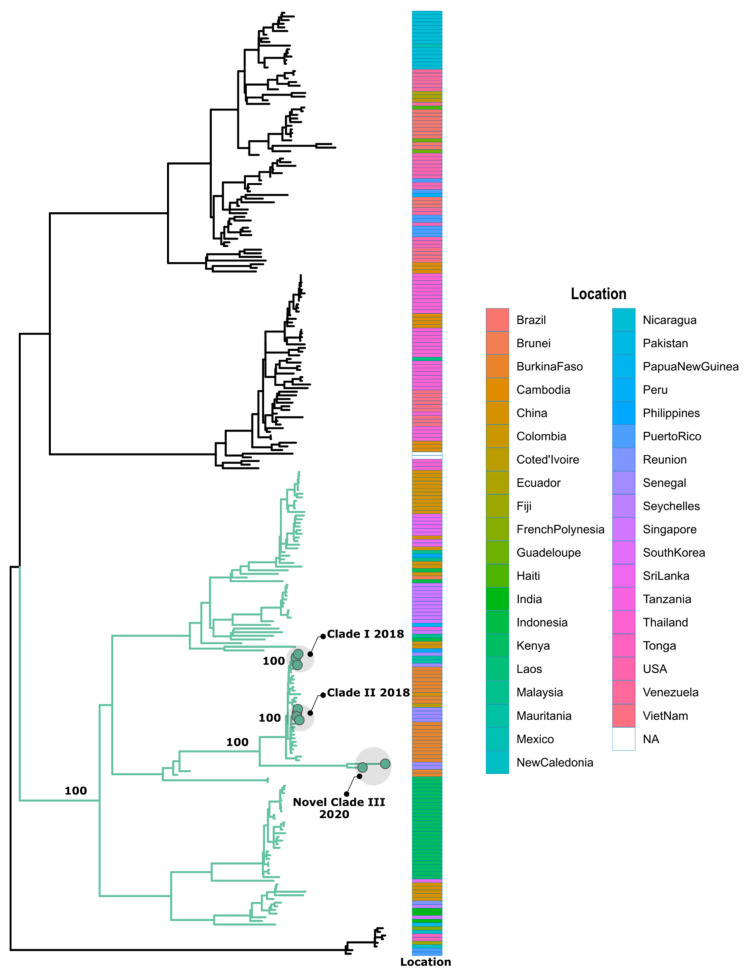
Maximum likelihood (ML) tree of DENV-2 in Senegal from January 2019 to February 2023. Branches of sequences belonging to the cosmopolitan genotype are colored in green. The analysis was based on 02 nearly complete genomes of DENV-2, generated during this study, in addition to n = 257 available sequences that were retrieved from VIPR database. The tree is midpoint-rooted. Study sequences harbor points on the sequence tip. Heatmap represents the location of collection of used DENV-1 sequences during phylogenetic analysis.

**Figure 7:**
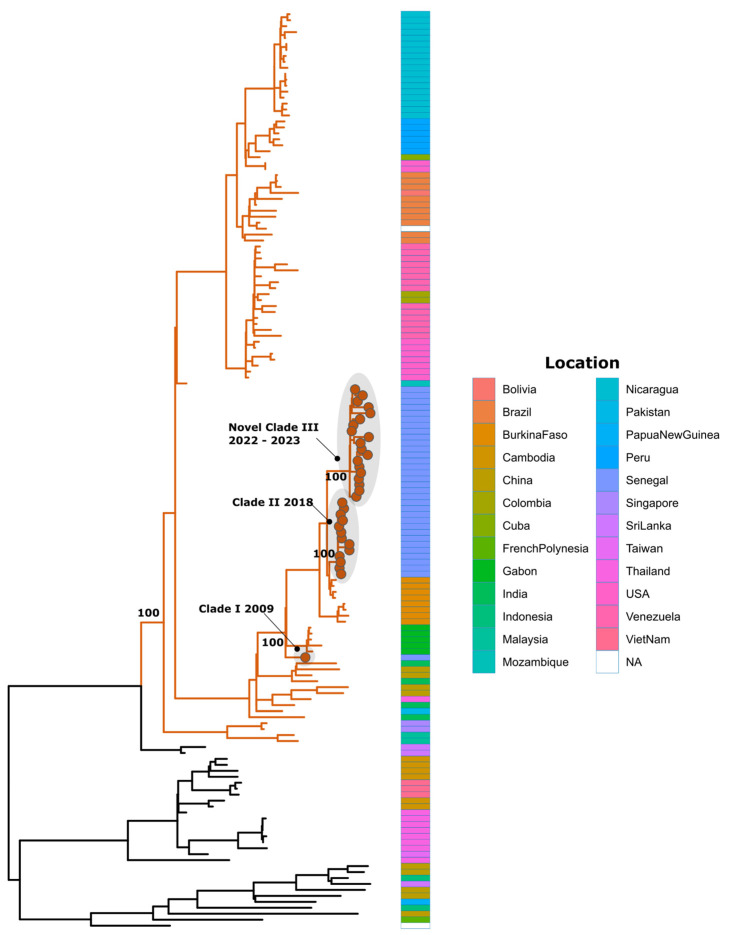
Maximum likelihood (ML) tree of DENV-3 in Senegal from January 2019 to February 2023. Branches of sequences belonging to genotype III are colored in orange. The analysis was based on 21 nearly complete genomes of DENV-3, generated during this study, in addition to n = 133 available sequences that were retrieved from VIPR database. The tree is midpoint-rooted. Study sequences harbor points on the sequence tip. Heatmap represents the location of collection of used DENV-1 sequences during phylogenetic analysis.

All these assignments were confirmed by phylogenetic analysis (Figure 5, Figure 6 and Figure 7). Additionally, phylogenetic analysis show that in comparison to limited previously available full genome sequences from Senegal DENV-1 and DENV-3 sequences clustered in different clades (hereafter, named Novel Clade II (2019–2021) for DENV-1 and Novel Clade III (2022–2023) for DENV-3 ; DENV-2 sequences are closely related to DENV-2 cosmopolitan, detected in West Africa with the emergence of Novel Clade III 2020.

## 4. Discussion

Senegal is a West African country with a reliable and efficient syndromic surveillance system, as exemplified by the previous early detections of epidemic-prone diseases and the subsequent organization of appropriate responses [12,43,44].

This system has allowed for the notification of many DENV outbreaks in Senegal [12,13,16]. Despite the recurrence of dengue epidemics, case studies focusing on the circulating serotypes/genotypes and their associated spatial and temporal distribution are limited [15]. This study aimed to address this concern by investigating the circulating dengue variants in Senegal between 2019 to 2023 through the syndromic sentinel surveillance network of Senegal (4S network). To the best of our knowledge, this study represents the first multiyear, countrywide study focusing on the temporal and spatial distribution of dengue virus serotypes/genotypes and viral genetic diversity using full genome sequences.

Among the suspected dengue samples (n = 5303), 402 were DENV RNA-positive (Figure 1). Interestingly, the confirmed cases were distributed around twelve out of the fourteen administrative regions of Senegal compared to seven regions during the 2017–2018 study [15], with a DENV RNA positivity rate of 7.58%. The highest number of dengue-positive cases was recorded in the Matam region with 166 cases, followed by Saint-Louis (n = 64), Dakar (n = 60), and Kaffrine (n = 29), and other regions where dengue was detected recorded a number of cases below twenty (Table S1). Interestingly, the Matam region has never been associated with any dengue outbreaks or recurrent case notifications in the past. This supports studies highlighting that the introduction of new groups of viruses to populations lacking prior exposure (serological naivety) has the potential to trigger unprecedented outbreaks and can be linked to more severe manifestations of dengue [27,45].

This is the largest study of dengue virus in Senegal, because of the large number of enrolled individuals. In contrast, a study on the genetic diversity of dengue virus in Bangkok yielded a higher prevalence of DENV positivity (25.09%) compared to our study [29], while a DENV RNA prevalence of 38.24% was obtained during a single-year study in India [46]. This discrepancy is probably due the fact that, compared to Senegal, dengue is highly endemic in Thailand and India. Indeed, Bangkok, the capital city of Thailand, is located in the center of the country and serves as a transportation hub, and dengue is known to circulate there since the 1950s [47,48]. In India, studies report that DENV is reported every year and the size, severity, and duration of outbreaks are increasing [46,49].

Taken together, our findings highlight the rapid spread of arboviral disease between neighboring regions due to travel and trade activities [50,51]. It is well known that frequent reintroductions of pathogens pose a significant challenge to elimination campaigns, especially in areas experiencing substantial regional and international travel. This is because humans serve as the reservoir host for both epidemic dengue and chikungunya [52]. No DENV+ RNA sample was recorded in the Sedhiou region in southern Senegal. This is probably due to the fact that the sentinel site in this region was implemented recently, and issues on proper sample transportation to IPD were noted. According to the year of collection, the highest number of confirmed DENV cases was in 2022. This trend follows the number of collected samples during the same year, which is higher compared to other years (Table 2). This is probably due to the fact that between 2019 and 2021, most of the surveillance efforts were focused on the COVID-19 pandemic.

For the assignment of DENV serotypes/genotypes, the only nationwide dengue spatial mapping study was based on partial CprM genes and used a limited number of samples, collected between 2017 and 2018 [15]. To get more up-to-date information on the genetic diversity of circulating dengue strains at the serotype/genotype levels, we serotyped 347 out of 402 dengue-positive samples by RT-qPCR and generated 34 nearly complete DENV genomes (Table 1). Serotyping using RT-qPCR showed that the detected dengue virus strains in Senegal during the study period belonged to DENV 1–3. The most represented serotype was DENV-3 (n = 276), followed by DENV = 1 (n = 66) and finally DENV-2 (n = 4). None of the DENV+ samples were linked to DENV-4 (Table S2). This finding corroborates those obtained by Dieng and colleagues, which showed the co-circulation of DENV 1–3 in Senegal between 2017 and 2018 [15].

Any marked spatial distribution pattern of serotypes was observed compared to a previous study in Senegal [15]. In contrast, a study performed in India showed a regional diversity of DENV serotypes [46].

Based on data from this study, DENV-3 was the dominant serotype in Dakar, Thies, Fatick, Diourbel, Kaolack, Kaffrine, Tambacounda, and Matam. DENV-1 was the dominant serotype in Saint-Louis. DENV-2 and DENV-3 were co-dominant in Louga (Figure 4A; Table S2). Multiple serotype infections were more prominent in Thies and Kaffrine, where DENV 1–3 were identified. In Africa, other countries, including Gabon and Burkina Faso, reported the co-circulation of at least three DENV serotypes [9,53]. This is probably linked to the fact that dengue surveillance and awareness is lacking on the continent [7]. The limited availability of data about DENV circulating serotypes/genotypes is due to the lack of national research institutions in many areas [49].

In contrast, studies in South America reveal the high-frequency co-circulation of dengue virus serotypes [54]. On the other hand, the hyperendemic nature of DENV virus epidemiology is well known and documented in Asian countries such as India [27,28,55] and in China, Malaysia, and Thailand [29].

Interestingly, the temporal trend of serotype circulation show that from the third week of the year in 2022, DENV-1 and DENV-2 serotypes were no longer circulating among confirmed DENV cases, but only DENV-3 was identified (Figure 3). This serotype shift was associated with a widespread and increased frequency of cases that were related to this serotype. These findings are similar those of Suzuki and colleagues in a study performed in Japan [56]. The fact that only DENV-3 was detected in Senegal may be due to an increased viral fitness of this serotype compared to DENV 1–2 [57]. Additionally, many other parameters such as a low level of herd immunity to the “new” serotype, the epidemic potential of the virus variants that are attributable to the serotype switch, and finally, the abundance and distribution of competent vectors can shape viral serotypes’ distribution and dynamic [17].

It is well known that different dengue virus serotypes can be associated with different phenotypic traits [58]. For instance, research conducted in Colombia examined the replicative capability of DENV within C6/36 mosquito cells and in populations of *A. aegypti*. This was carried out using a distinct viral strain for each DENV serotype, revealing varying degrees of fitness among the serotypes [58]. Besides intra-serotypic genetic diversification, other parameters such as cross-protective immunity between serotypes may explain this observed DENV serotype replacement phenomenon [59]. The hypothesis about differential viral fitness should be tested by in vitro and in vivo studies, since vector-driven selection may contribute to a viral replacement phenomenon, as described previously in New Caledonia [22].

A phylogenetic analysis, as well as genotyping using Genome Detective dengue typing tools, show that the genotype diversity of the detected DENV serotypes was relatively low in our study. Indeed, each serotype consisted of a single genotype. The DENV-1 strains belonged to genotype V, DENV-2 strains to the cosmopolitan genotype, and finally, DENV-3 strains to genotype III. This trend is comparable to the genotypic dengue virus make-up that is found in Africa [24]. However, the genetic diversity of Senegalese DENV strains was more pronounced within each genotype. Indeed, each genotype of the characterized DENV sequences falls into different Clades. DENV-1 sequences were distributed in two separate Clades, namely, Clade I 2018, composed of viruses sampled during the 2018 outbreak in the Thies regions [14], and Novel Clade II 2019–2021, including strains that were principally associated to the outbreak in Rosso in 2021 [60], in addition to sporadic cases collected in late 2019 and the end of 2021. The same trend was observed for DENV-3, with the observations of the circulation of contemporary viruses belonging to two clades: Clade II 2018, shared with strains circulating in Thies in 2018 [14] and in Senegal 2019, in addition to a newly identified Novel Clade III 2022–2023, which is closely related to viruses sampled in Burkina Faso in 2017 and Ethiopia in 2019. The occurrence of viral strains belonging to different Clades reflects the extent of our sampling and is a hallmark of different origins of transmission. These results call for in-depth phylogeographic studies to elucidate their origin and dispersal patterns.

In general, the presence of various strains from different serotypes/genotypes/clades could potentially account for the consistent reports of dengue outbreaks within the country since 2017. Collectively, these findings underscore the vital importance of maintaining an ongoing genomic surveillance system for DENV in Senegal. The data generated through this surveillance system can be utilized to support public health laboratories in monitoring the diversity of the virus, which is essential for implementing effective control measures.

Overall, this study evaluates for the first time in Senegal the distribution of DENV variants (serotypes/genotypes) using whole-genome sequencing. This constitutes an important and relevant public health effort to decipher the virus’s molecular epidemiology. Despite the importance, some limitations associated with the study need to be mentioned. First, during the study, all samples were transiently stored at +4 on site before shipping to the reference lab; this can affect the viral RNA integrity, which is crucial for molecular detection. Second, the described diversity reflects the extend of samples that are subjected to sequencing; the selection of samples to be sequenced can be influenced by a sampling bias.

## 5. Conclusions

In summary, it is essential to maintain ongoing monitoring of the circulating DENV serotypes/genotypes in Senegal. This ongoing surveillance will provide crucial information to guide proactive and well-informed public health interventions. By remaining vigilant and adaptable in the face of viral variants, we can effectively navigate and respond to emerging waves of infections, minimizing their impact and safeguarding the health of the population. Consistent genomic surveillance coupled with real-time data analysis offer invaluable insights into the evolutionary dynamics of the virus. This, in turn, aids in making informed decisions for public health responses. Comprehending the patterns of circulation of these DENV variants contributes to a comprehensive understanding of the virus’s current status in a local/regional context. This enables authorities to implement appropriate measures, including refining testing strategies. These measures effectively mitigate the impact of new infection waves and prevent rapid spread within communities. In the light of this work, continuous monitoring of DENV serotypes/genotypes in Senegal is thus warranted to achieve better control of the virus and the development of effective vaccines in endemic areas.

## Figures and Tables

**Table 1:** Summary of sequenced DENV samples during this study.

ID	Virus Type	Genotype	Region	Isolate	Sample Type	Collection Date	Depth of Coverage	Genome Coverage (%)
**SH 377553**	DENV-1	Genotype V	Saint-Louis	Human	Serum	29 October 2021	1354.2	91.2
**SH 377552**	DENV-1	Genotype V	Saint-Louis	Human	Serum	29 October 2021	3207.7	91.2
**SH 377551**	DENV-1	Genotype V	Saint-Louis	Human	Serum	28 October 2021	162.9	58.3
**SH 377538**	DENV-1	Genotype V	Saint-Louis	Human	Serum	1 November 2021	2043.9	89.4
**SH 377555**	DENV-1	Genotype V	Saint-Louis	Human	Serum	29 October 2021	6465	93.7
**SH 377554**	DENV-1	Genotype V	Saint-Louis	Human	Serum	29 October 2021	3079	93.6
**SH 322838**	DENV-1	Genotype V	Dakar	Human	Serum	21 October 2019	1762.7	93.2
**SH 322765**	DENV-1	Genotype V	Dakar	Human	Serum	15 October 2021	2583.8	93.4
**SH 318479**	DENV-1	Genotype V	Louga	Human	Serum	24 January 2019	1045.8	88.8
**SH 318478**	DENV-1	Genotype V	Louga	Human	Serum	24 January 2019	2372.3	93.7
**SH 318267**	DENV-1	Genotype V	Louga	Human	Serum	10 January 2019	4266	93.6
**SH 326097**	DENV-2	Genotype II	Kaffrine	Human	Serum	30 September 2020	5691.0	75.6
**SH 323592**	DENV-2	Genotype II	Thies	Human	Serum	5 January 2020	4893.3	81.5
**SH 377545**	DENV-3	Genotype III	Saint-Louis	Human	Serum	27 October 2021	6991.1	94.7
**SH 377540**	DENV-3	Genotype III	Saint-Louis	Human	Serum	26 October 2021	2533.1	89.4
**SH 377524**	DENV-3	Genotype III	Thies	Human	Serum	28 October 2021	5318	98
**SH 377522**	DENV-3	Genotype III	Thies	Human	Serum	28 October 2021	4902.2	96.7
SH 392244	DENV-3	Genotype III	NA	Human	Serum	NA	2670	93.4
**SH 402404**	DENV-3	Genotype III	Fatick	Human	Serum	22 August 2022	4915	93.4
**SH 392518**	DENV-3	Genotype III	Tambacounda	Human	Serum	19 March 2022	5708.4	94.8
**SH 392408**	DENV-3	Genotype III	Matam	Human	Serum	15 March 2022	3506.2	93.4
**SH 392406**	DENV-3	Genotype III	Matam	Human	Serum	15 March 2022	3946.5	93.4
**SH 392265**	DENV-3	Genotype III	Matam	Human	Serum	25 February 2022	4319.5	95.7
**SH 392263**	DENV-3	Genotype III	Matam	Human	Serum	25 February 2022	3527.8	93.4
**SH 392260**	DENV-3	Genotype III	Matam	Human	Serum	23 February 2022	3906.5	96.4
**SH 392169**	DENV-3	Genotype III	Matam	Human	Serum	22 February 2022	4515	94.7
**SH 392168**	DENV-3	Genotype III	Matam	Human	Serum	21 February 2022	5482.1	95.9
**SH 330006**	DENV-3	Genotype III	Dakar	Human	Serum	3 November 2020	5740.9	93.6
**SH 330004**	DENV-3	Genotype III	Dakar	Human	Serum	3 November 2020	2271.8	71.7
**SH 329067**	DENV-3	Genotype III	NA	Human	Serum	NA	1966.4	91.6
**SH 327002**	DENV-3	Genotype III	Dakar	Human	Serum	12 October 2020	2597.6	89.8
**SH 392572**	DENV-3	Genotype III	Matam	Human	Serum	21 March 2022	6.4	52.4
**SH 392571**	DENV-3	Genotype III	Matam	Human	Serum	22 March 2022	5843.5	95.9
**SH 323342**	DENV-3	Genotype III	Kaffrine	Human	Serum	10 December 2019	10,262.1	95
**SH 322872**	DENV-3	Genotype III	Kaffrine	Human	Serum	28 October 2019	6151.3	94.4

**Table 2:** Repartition of suspected and confirmed DENV cases according to the year of sampling.

Year of Collection	Number of Suspected Cases	Number of Confirmed DENV Cases	Positivity Rate (%)
**2019**	890	19	2.13
**2020**	862	20	2.32
**2021**	1353	131	9.68
**2022**	2089	216	10.33
2023 **	109	15	13.76

** Surveillance data for only two months (January to February) of 2023 were considered during this study.

## Data Availability

The data that support the findings of this study are available from the corresponding author upon reasonable request.

## Supplementary Materials

The following supporting information can be downloaded at https://www.mdpi.com/article/10.3390/tropicalmed9020032/s1: Table S1: Number of DENV-positive samples recorded per region from 2019 to 2023 through the 4S network; Table S2: Summary of the number of serotyped samples at each monitoring region; Table S3: Reparation of detected DENV RNA-positive samples per year and week from 2019 to 2023; Table S4: Reparation of detected DENV serotypes per year week from 2019 to 2023.
