# Supplementary material for: The Spatiotemporal Distribution and Molecular Characterization of Circulating Dengue Virus Serotypes/Genotypes in Senegal from 2019 to 2023
Source: Trop Med Infect Dis. 2024 Jan 27;9(2):32. doi: 10.3390/tropicalmed9020032 (PMC10891755; doi:10.3390/tropicalmed9020032)
Supplement: Supplementary file 1 [file tropicalmed-09-00032-s001.zip › tropicalmed-2833199-supplementary.pdf]

Table S1: Number of DENV positives samples recorded per regions from 2019 to 2023 through the 4S network.

Region	Number of DENV positive samples
Dakar	60
Diourbel	06
Fatick	08
Kaffrine	29
Kaolack	16
Kedougou	01
Kolda	01
Louga	06
Matam	166
Saint-Louis	64
Tambacounda	05
Thies	39
Ziguinchor	01

Table S2 : Summary of the number of serotyped samples at each monitoring region

Region	DENV-1	DENV-2	DENV-3
Dakar	04	00	44
Thies	01	01	32
Fatick	00	00	04
Diourbel	00	00	04
Kaolack	00	01	13
Kaffrine	01	02	20
Saint Louis	53	00	03
Louga	03	00	03
Tambacounda	00	00	02
Matam	04	00	151
**Total**	66	04	276

Table S3: Reparation of detected DENV RNA positive samples per Year_Week from 2019 to 2023

Year-Week	Number of Cases
2019-02	1
2019-04	2
2019-33	1
2019-39	1
2019-42	2
2019-43	2
2019-44	2
2019-45	3
2019-46	1
2019-47	2
2019-49	1
2019-50	1
2020-01	1
2020-28	1
2020-38	1
2020-40	1
2020-41	1
2020-42	1
2020-43	3
2020-44	3
2020-45	1
2020-46	3
2020-47	2
2020-48	1
2020-49	1
2021-04	1
2021-05	1
2021-36	1
2021-39	7
2021-40	7
2021-41	16
2021-42	8
2021-43	21
2021-44	18
2021-45	11
2021-46	7
2021-47	11
2021-48	5
2021-49	3
2021-50	3
2021-51	10
2021-52	1
2022-01	4
2022-02	1
2022-03	3
2022-04	9
2022-05	5

2022-06	2
2022-07	6
2022-08	6
2022-11	2
2022-12	5
2022-13	4
2022-14	1
2022-16	1
2022-34	1
2022-35	1
2022-36	2
2022-39	2
2022-40	3
2022-41	4
2022-42	5
2022-43	9
2022-44	9
2022-45	13
2022-46	17
2022-47	20
2022-48	22
2022-49	20
2022-50	14
2022-51	18
2022-52	7
2023-01	2
2023-02	4
2023-03	2
2023-04	2
2023-05	2
2023-06	2
2023-07	1

Table S4: Reparation of detected DENV serotypes per Year_Week from 2019 to 2023

Years_Week	DENV-1	DENV-2	DENV-3
2019-02	1	0	0
2019-04	2	0	0
2019-33	0	1	0
2019-39	0	0	1
2019-42	2	0	0
2019-43	0	0	2
2019-44	0	0	1
2019-45	0	0	2
2019-46	0	0	0
2019-47	0	0	0
2019-49	0	0	0
2019-50	0	0	1
2020-01	0	1	0
2020-28	0	0	0
2020-38	0	1	0
2020-40	0	1	0
2020-41	0	0	1
2020-42	0	0	0
2020-43	0	0	0
2020-44	0	0	2
2020-45	0	0	0
2020-46	0	0	0
2020-47	0	0	0
2020-48	0	0	0
2020-49	0	0	0
2021-04	0	0	0
2021-05	0	0	0
2021-36	0	0	1
2021-39	7	0	0
2021-40	5	0	2
2021-41	11	0	1
2021-42	7	0	0
2021-43	14	0	6
2021-44	11	0	2
2021-45	1	0	9
2021-46	0	0	3
2021-47	1	0	7
2021-48	1	0	3
2021-49	1	0	2

2021-50	0	0	2
2021-51	1	0	8
2021-52	0	0	1
2022-01	0	0	4
2022-02	0	0	1
2022-03	1	0	2
2022-04	0	0	9
2022-05	0	0	3
2022-06	0	0	0
2022-07	0	0	5
2022-08	0	0	5
2022-11	0	0	2
2022-12	0	0	5
2022-13	0	0	4
2022-14	0	0	0
2022-16	0	0	0
2022-34	0	0	1
2022-35	0	0	1
2022-36	0	0	2
2022-39	0	0	2
2022-40	0	0	3
2022-41	0	0	4
2022-42	0	0	4
2022-43	0	0	8
2022-44	0	0	8
2022-45	0	0	13
2022-46	0	0	17
2022-47	0	0	20
2022-48	0	0	22
2022-49	0	0	20
2022-50	0	0	14
2022-51	0	0	18
2022-52	0	0	7
2023-01	0	0	2
2023-02	0	0	4
2023-03	0	0	2
2023-04	0	0	2
2023-05	0	0	2
2023-06	0	0	2
2023-07	0	0	1
